# Influence of Chlorhexidine and Cetylpyridine on Periodontal Status and Indicators of Oxidative Stress in Patients with Type 1 Diabetes

**DOI:** 10.3390/antiox10111732

**Published:** 2021-10-29

**Authors:** Jakub Lipski, Anna Duda-Sobczak, Marta Napierala, Ewa Florek, Dorota Zozulinska-Ziolkiewicz, Marzena Wyganowska-Swiatkowska

**Affiliations:** 1 Department of Dental Surgery and Periodontology, Poznan University of Medical Sciences, 60-812 Poznan, Poland; kuba.lipski@gmail.com (J.L.); marzena.wyganowska@periona.pl (M.W.-S.); 2 Department of Internal Medicine and Diabetology, Poznan University of Medical Sciences, 60-834 Poznan, Poland; zozula@box43.pl; 3 Department of Toxicology, Poznan University of Medical Sciences, 60-631 Poznan, Poland; martan@ump.edu.pl (M.N.); eflorek@ump.edu.pl (E.F.)

**Keywords:** chlorhexidine digluconate, cetylpyridine chloride, oxidative stress, type 1 diabetes

## Abstract

Objective: One of the treatment goals in type 1 diabetes and periodontitis is to address chronic inflammation to prevent the development of neurovascular complications. The aim of this study was to assess the local anti-inflammatory effects of chlorhexidine digluconate and cetylpyridine chloride on periodontal status and indicators of oxidative stress in saliva in patients with type 1 diabetes. Materials and Methods: A total of 42 subjects aged 27 (interquartile range, IQR 22–35) years, with type 1 diabetes for a duration of 12 (IQR 9–18) years, and glycated hemoglobin 8.05 (IQR 7.1–9.4)% were included. Patients were examined twice—initially, and after 14 days of using toothpaste with chlorhexidine and cetylpyridine. Clinical examination of gingival tissues was performed. Certain oxidative stress markers (TP, TEAC, TBARS, AOPP) were measured in the saliva samples. Results: There were significant changes in clinical indicators of periodontal status before and after the application of the toothpaste (API before 0.35 (0.24–0.65) vs. API after 0.265 (0.18–0.39), *p* = 0.03; SBI before 0.07 (0.04–0.15) vs. SBI after 0.035 (0-0.06), *p* = 0.002; GI before 0.88 (0.46–1) vs. GI after 0.67 (0.25–1), *p* = 0.0008). The concentration of saliva TBARS decreased (*p* = 0.00005) and TEAC increased (*p* = 0.09). Conclusion: Proper oral hygiene supported by antibacterial chemicals may improve the periodontal status and reduce inflammation.

## 1. Introduction

The basis of type 1 diabetes (T1D) is an autoimmune inflammation leading to destruction of the β cells of the pancreatic islets that produce insulin [1]. Chronic hyperglycemia and acute glucose variations are associated with low-grade inflammation and oxidative stress [2]. Several pathogenic pathways are involved [3]. These include activation of the protein kinase C pathway, formation of glycation end-products (AGEs), accumulation of sorbitol through the aldose reductase pathway, and activation of the hexosamine pathway and the polyADP-ribose polymerase (PARP) pathway, which regulates several inflammatory response gene expressions [4]. The duration of hyperglycemia, acting as a cell and extracellular matrix proliferative factor, results in the exacerbation of oxidative and nitrative stress, further inflammatory response, insulin resistance, and protein glycation [5], and it clinically manifests as micro and macrovascular dysfunction and the development of retinopathy, chronic kidney disease, neuropathy and periodontal disease [6]. A generation of reactive oxygen species (ROS) has been proposed as the essential element of different pathogenic pathways [3]. The β cells are especially sensitive to reactive oxygen species (ROS), which includes free radicals and non-radical species [7].

Through oxidation of the thiol group, ROS can directly activate all molecules containing sulfhydryl groups, such as proteins or glutathione (GSH), and through the oxidation of proteins, nucleic acids and polyunsaturated fatty acids react with cell components [8]. The best known free-radical process is lipid peroxidation, a non-enzymatic process in which the oxidation of unsaturated fatty acids takes place with the formation of peroxides of these compounds [9,10]. The production of aldehydes such as dimalonic hydroxyaldehyde (MDA), though a non-specific marker of lipid peroxidation, is particularly dangerous [11]. It is a laboratory index of oxidative stress in the reaction with thiobarbituric acid (TBARs) [12,13]. Due to the ease of combining MDA with collagen and elastin, in patients with diabetes, it is associated with stiffening of the vessel walls [14]. Additionally, circulating LDLs in the blood—mainly unsaturated fatty acids of triacylglycerols and cholesterol esters—are readily oxidized by ROS [15]. Oxidized LDL (oxLDL: oxidized low-density lipoproteins), through their influence on the cell cycle, lead to endothelial dysfunction, which results in cell proliferation and hyperplasia, as well as apoptosis [16]. Macrophages capture oxLDL and transform them into foam cells, which are the fundamental elements of early atherosclerotic lesions [17].

The modification of prosthetic groups and amino acid residues in proteins, and the aggregation or fragmentation of protein molecules, is the result of oxidative damage [18]. The action of ROS on proteins relates to the damaging action of several amino acid residues, including histidine, proline, arginine and lysine, leading to the formation of carbonyl groups [18]. Oxidative damage to proteins leads to rapid loss of their biological activity. Laboratory markers of protein oxidative damage are nitrotyrosine, methionine sulfoxide (MetSO), *N*-formylokinurenine (NFK) and the concentration of end products of protein oxidation (AOPP) [19]. It should be noted that the peroxidation of proteins and nucleic acids is not chain-like in lipids. Oxidized proteins and nucleic acids more often react with low molecular weight antioxidants, lowering the pool of these compounds [20,21].

Our previous study showed that infection in periodontal tissue disease might be one of the clinical manifestations of chronic inflammation in type 1 diabetes [22,23,24]. It is also well established that chronic hyperglycemia and oxidative stress lead to an imbalance between the opportunistic bacteria of biofilm and the host immune response in the oral cavity environment, which results in inflammation and the possible destruction of periodontal tissue [23,24,25]. On the other hand, the chronic infection, with the activation of components of the immune system, in periodontal tissue is the source of free radicals [26]. The gold standard in controlling the periopathogens in biofilm is mechanical debridement supported by mouth rinsing with chemical agents [27]. Chlorhexidine (1:6-Di 4’-chlorophenyl-diguanido hexane) (CHX), used as the chlorhexidine gluconate, is a synthetic antimicrobial agent that is highly effective against a wide range of microorganisms [28]. Cetylpyridinium chloride (CPC) is a quaternary ammonium compound, which has moderate plaque inhibitory activity and antibacterial activity equivalent to CHX [29].

The aim of this study was to assess the effect of chlorhexidine digluconate and cetylpyridine chloride on the clinical condition of the periodontium and the indicators of oxidative stress in saliva in patients with type 1 diabetes.

## 2. Materials and Methods

### 2.1. Clinical Examination

This study was conducted on patients with T1D under the care of the Department of Internal Medicine and Diabetology in cooperation with the Department of Dental Surgery and Periodontology and the Department of Toxicology, Poznan University of Medical Sciences between 2018 and 2020. It was approved by the local ethics committee (No 1066/15, 4 February 2016) and conducted according to the guidelines of the Declaration of Helsinki on biomedical research involving human subjects. All participants provided written informed consent before enrollment in the study. Of 65 patients, 42 were included in the examination. Exclusion criteria were a diabetes duration of less than 5 years, an age above 40 years, diabetic ketoacidosis at the time of enrollment, pregnancy and, in the case of one patient, a lack of two properly collected samples of saliva. A diabetologist was responsible for the enrollment process. All patients underwent a complete physical examination with anthropometric measurements.

The collection of venous blood samples (using a standard venipuncture) was performed at the beginning of the study in the morning between 8:00 and 10:00 am by the S-Monovette blood collection system (Sarstedt, Aktiengesellschaft & Co., Numbrecht, Germany) after at least a 12 h fast. The following techniques were applied in laboratory measurements: competitive turbidimetric inhibition immunoassay (TINIA) with the twin test reaction mode for HbA1c on a Cobas analyzer (Roche Diagnostics, Basel, Switzerland) according to DCCT/NGSP (DCCT, Diabetes Control and Complication Trial; NGSP, National Glycohemoglobin Standardization Program) [30] and commercially available enzymatic assays (Roche Diagnostics, Basel, Switzerland) for: high-density lipoprotein (HDL), low-density lipoprotein (LDL), triglycerides (TG), total cholesterol (TC), and highly specific C-reactive protein (hsCRP).

The saliva was collected twice, by the periodontologist, at the beginning of the study and after 2 weeks of tooth brushing with GUM Paroex toothpaste containing 0.06% chlorhexidine digluconate + 0.05% cetylpyridinium chloride with at least two toothbrushing sessions lasting a minimum of 2 min per day. During this time, patients were asked to not change their oral hygiene habits. The first examination of periodontal status in all patients who were included in the examination was considered as a control group. Periodontal status was also examined during the same time. To avoid potential bias, one periodontologist assessed periodontal health with a WHO perio probe. The gingival index (GI) according to Silness and Loe [31] and the approximal plaque index according to Lange were assessed to determine the hygienic status of patients [32], in quadrant 1 and the oral cavity, and in quadrants 2 and 4 from the side vestibule. These indexes are simple indicators to use in an outpatient setting, assessing the presence of tiles on the contact surfaces where they form. The assessment was made in a zero-single. Change in the number of deposits expressed as a percentage is the basis for the assessment of the effectiveness of hygiene treatments. The individual ranges of the indicator have been interpreted according to the following division: API < 25%-optimal hygiene; API = 25–39%-almost good hygiene; API = 40–70%-average hygiene, to be improved; API = 70–100%-improper hygiene. In the same quadrants but on the opposite site, according to Muhlemann and Son, the sulcus bleeding index was used to check the activity of the inflammatory process in the periodontium [33]. Before collecting saliva into a Salivette tube, the patient rinsed their mouth with deionized water. Samples of saliva were always taken simultaneously and the patient did not eat for at least 6 h prior to testing. After collection, the samples were immediately transported to the Department of Toxicology for oxidative stress indication.

### 2.2. Determination of Oxidative Stress Markers

Relevant markers of oxidative stress and biochemical parameters (total protein concentration, TP; the trolox equivalent antioxidant capacity, TEAC; thiobarbituric acid reactive substances, TBARS; the advanced oxidation protein products, AOPP) were determined in saliva using spectrophotometric methods.

A quantification of the total protein concentration (TP) was performed with the use of the Lowry et al. method based on a sensitive reaction of peptide bonds and aromatic amino acids with the Folin–Ciocalteu reagent [34].

The trolox equivalent antioxidant capacity (TEAC) of substances present in the solutions was measured based on the measurement of the stable radical cation reduction capacity, the pre-formed radical monocation of 2,2′-azinobis-(3-ethylbenzothiazoline-6-sulfonic acid) (ABTS)^•+^ [35].

A thiobarbituric acid reactive substances (TBARS) measurement was used for monitoring lipid peroxidation [36].

The advanced oxidation protein products (AOPP) assay is a method to determine oxidative stress, based on a spectrophotometric assay that uses the ability to create colored reaction products of potassium iodide with the products of protein oxidation in an acidic environment. The concentration of protein oxidation products was read using a standard curve for the reaction of potassium iodide with chloramine T [37].

### 2.3. Statistics

Statistica PL version 13.0 (Statsoft, Tusla, OK, USA) was used for the statistical analysis of the obtained data. The significance level *p* < 0.05 was considered statistically significant. The Kolmogorov–Smirnov test showed that the distribution of the obtained data differed in a statistically significant way from the normal distribution. Therefore, non-parametric tests were used for further data analysis. The Mann–Whitney U test and the Wilcoxon signed-rank test were used in the statistical analysis. The results are presented as median and interquartile range (IQR).

## 3. Results

The median age of the included participants was 27 (interquartile range, IQR 22–35) years and the mean diabetes duration was 12 (IQR 9–18) years. The metabolic control of diabetes expressed by HbA1c was not optimal as the target value for HbA1c in type 1 diabetes is below 6.5%However, he target may be modified in certain individuals (Table 1). According to the recommendations of the American Diabetes Association, the appropriate general HbA1c goal for many nonpregnant adults is <7%. On the basis of provider judgment and patient preference, the achievement of lower HbA1c levels than the goal of 7% may be acceptable, and even beneficial, if it can be achieved safely without significant hypoglycemia or other adverse effects of treatment. Less stringent HbA1c goals (such as <8%) may be appropriate for patients with limited life expectancy, or where the harms of treatment are greater than the benefits [38]. The studied group was not obese (no subjects with body mass index, BMI ≥ 30 kg/m^2^); however, some subjects were overweight (BMI ≥ 25, but <30 kg/m^2^). The risk for the development of chronic complications of diabetes correlates with the metabolic control of the disease and rises with the rise of the level of glycated hemoglobin [39]. We therefore subdivided study group into two groups according to the median HbA1c (we assumed HbA1c 8% for the division threshold). There were no differences in age, diabetes duration, C-reactive protein, total cholesterol, LDL-cholesterol and HDL-cholesterol between subgroups. The HbA1c > 8% group had significantly higher triglyceride levels (*p* = 0.01) (Table 2). There were significant changes in clinical indicators of periodontal status before and after the application of the toothpaste (API before 0.35 (0.24–0.65) vs. API after 0.265 (0.18–0.39), *p* = 0.03; SBI before 0.07 (0.04–0.15) vs. SBI after 0.035 (0–0.06), *p* = 0.002; GI before 0.88 (0.46–1) vs. GI after 0.67 (0.25–1), *p* = 0.0008) (Table 3). Those changes were still significant in the analysis of subgroups of 8% ≤ HbA1c > 8%, except API in HbA1c > 8% group (Table 4).

We analyzed the concentration of selected oxidative stress indicators in saliva before and after the application of the toothpaste (Table 5). The concentrations of saliva protein and AOPP were lower after toothpaste use but did not reach statistical significance. However, the concentration of saliva TBARS decreased (*p* = 0.00005) and TEAC increased (*p* = 0.09) significantly. Those changes were significant in the analysis of subgroups of 8% ≤ HbA1c > 8% for TBARS, and TEAC changed significantly only in HbA1c > 8% group (Table 6).

As the general target for HbA1c in diabetes is 7%, we performed additional analysis comparing subgroups with HbA1c ≤ 7% and HbA1c > 7%. Interestingly, there were no differences in clinical indicators of periodontal status before and after the application of toothpaste in HbA1c ≤ 7%, although there was still significant improvement in periodontal status in HbA1c > 7% (API before 0.35 (0.25–0.65) vs. API after 0.28 (0.18–0.39), *p* = 0.003; SBI before 0.065 (0.03–1.12) vs. SBI after 0.035 (0–0.06), *p* = 0.02; GI before 0.83 (0.46–1) vs. GI after 0.67 (0.25–1), *p* = 0.01). The concentration of TBARS decreased significantly in both HbA1c ≤ 7% and HbA1c>7% subgroups (*p* = 0.01 and *p* = 0.002, respectively) and the concentration of TEAC increased significantly in HbA1c > 7% group (*p* = 0.03).

## 4. Discussion

The toothpaste used in the study is one of only a few available that contains two of the most effective antimicrobial chemical agents, 0.06% chlorhexidine digluconate + 0.05% cetylpyridinium chloride. This combination and coworking is able to significantly reduce by approximately 60% the metabolic activities of biofilm and to reduce by approximately 96–99% of the total bacteria counts [40]. It is suggested that combining CHX and CPC allows for reducing the chemical agents’ concentration, thereby lowering their side effects, such as tooth discoloration, mouth burning and cytotoxicity [41,42]. The results of our study confirm the efficiency of the toothpaste on the clinical condition of periodontal tissue in type 1 diabetes patients after a two-week period of brushing and suggest that CHX with CPC may be an effective inhibitor of biofilm colonizers and may not disturb a healthy gingiva. This is in agreement with another study [43]. It is important that patients did not change their habits during examination time. The values of all clinical indexes connected with oral hygiene and clinical symptoms of inflammation were statistically lower after examined toothpaste use. Interestingly, the influence of toothpaste on the concentration of oxidative stress indicators in saliva was most effective in poorly controlled patients, with higher HbA1c. There was no significant change according to periodontal indices in subjects with HbA1c ≤ 7%, an accepted general indicator of good diabetic control. Further analysis of subgroups with HbA1c below and over 8% showed significant changes in periodontal indices of inflammation in both groups. This may suggest that anti-inflammation intervention is clinically effective in subjects who are not reaching a general target of good metabolic control. However, control of the hygiene level (API) was not significantly improved in subjects with HbA1c > 8%. Chronic hyperglycemia, glucose fluctuations and high HbA1c are associated with the development of neurovascular complications which clinically manifest as retinopathy, renal failure or neuropathy [44]. In those conditions, several pathophysiological metabolic pathways are being activated [3,45]. Experimental studies show that the properties of many cells change under the influence of concentrated glucose solutions, especially endothelial cells, leukocytes and platelets [46]. The glucose transporting protein GLUT 1 is involved in glucose influx into those cells, and its activity is not down-regulated due to changes in glucose concentrations [47]. As a result, any fluctuations in blood glucose concentration lead to disturbances of its intracellular transport and metabolism [48]. Within cells, glucose metabolism occurs through several metabolic pathways (glycolytic, pentose, hexosamine and polyol pathways). Under conditions of hyperglycemia, the intensification of glucose metabolism in endothelial cells, granulocytes, monocytes and platelets is accompanied by an increased production of reactive oxygen species, ROS [49]. Studies attempt to use markers of oxidative stress as additional clinical markers for assessing metabolic control of diabetes and for determining the risk of developing micro- and macroangiopathy [50,51]. Proper treatment of diabetes and achieving target glucose values enable patients to lower the concentration of selected oxidative stress markers [52]. Additionally, the accumulation of advanced glycation-end products (AGEs) and lipid peroxidation contribute to the risk of developing chronic complications of diabetes. AGEs are heterogeneous compounds mainly derived from the nonenzymatic glycation (Maillard reactions) of reducing sugar on proteins, lipids, and nucleic acids. AGEs can increase reactive oxygen species (ROS) production, thereby initiating intracellular oxidative stress [22]. Conversely, the increase in ROS production can promote the production of AGEs, thereby forming a vicious circle between oxidative stress and AGEs [53]. Lipid peroxidation is a non-enzymatic process controlled by antioxidant mechanisms, in particular by the enzyme system associated with SeGSH-Px (selenium-dependent glutathione peroxidase) [54]. Especially dangerous is the production of aldehydes such as dimalonic hydroxyaldehyde (MDA) MDA is a non-specific marker of lipid peroxidation, and laboratory marker of oxidative stress in the reaction with thiobarbituric acid (TBARs) [12]. In turn, oxidative damage of proteins leads to a rapid loss of their biological activity. Laboratory markers of protein oxidative damage are: nitrotyrosine [20], methionine sulfoxide (MetSO), N-form-quinurenine (NFK) [55] and the concentration of end products of protein oxidation (AOPP). It should be noted that the peroxidation of proteins and nucleic acids is not chained in nature, as is the case with lipids. The activity of a peroxidase in the presence of hydrogen peroxide and 2,2’-azobis-(2-amidinopropane) results in radical cation 2,2′-azinobis-(3-ethylbenzothiazoline-6-sulfonate) (ABTS^•+^) evaluated in trolox equivalent (TEAC) measurements [56]. In our study, the decrease in both TEAC and TBARs after tooth cleaning was statistically important.

There are two peroxidases detected in the saliva: lactoperoxidase, produced by the parotid and submandibular glands; and myeloperoxidase, contained in polymorphonuclear neutrophils [57]. Both enzymes also exhibit an antimicrobial potential through oxidation of the thiocyanate ion [57], which restricts periopathogen proliferation in the mouth by oxidizing thiol residues in essential microbial proteins [58]. The lowering value of TEAC in saliva seems to be naturally connected with reducing the number of bacteria in the oral microbiome. However, the microbiome composition fluctuates during the day and is also connected with diet [59]. It is well known that the oral microbiome is a complex environment in which pathogens influence each other; some of them have the ability to limit the oxidative stress effect by producing antioxidant enzymes; others produce ROS to limit the growth of other species and occupy the ecological niche [60].

Saliva, especially parotid saliva, can also reduce peroxide of fatty acids, and thereby have a role in the protection against lipid peroxidation [61]. It was found that higher total cholesterol was not connected with periodontal disease; however, higher triglyceride levels were [62]. In our study, only the level of triglycerides correlated positively with higher level of HbA1c. However, it is possible that this correlation changes with age. In the group of T1D children aged 7-13, all lipid parameters were higher; however, statistical difference was observed only for HDL, triglycerides and total cholesterol [63].

Furthermore, some periopathogens have been found to be associated with diverse lipid metabolites. *T. forsythia* and *P. gingivalis* had a significant association with HDL-35, while serum IgG1 against *T. forsythia* was associated with triglycerides [64]. The current study also pointed to Galcectin-3, NLRP3, IL-1b as an additional biomarker in crevicular fluid, which controls the progress of periodontal disease [65,66,67].

Our study has several limitations that should be mentioned. The chosen 14-day period of toothpaste use was based on the authors’ experience, as there are no guidelines on duration and recommended frequency of toothpaste use. We were also not able to verify the participants’ adherence to regular toothpaste use, which might influence the results. Finally, the single-center outreach of our study might be a flaw.

## 5. Conclusions

Proper oral hygiene supported by appropriate agents with antibacterial features may positively affect the periodontal status, reducing the indicators of periodontal inflammation and oxidative stress in subjects with type 1 diabetes when the HbA1c is tightly controlled. The increasing HbA1c levels influence the value of those indexes (SBI, GI), which are connected with the inflammation process relevant to microvascular complications typical for diabetes. Further research is needed to assess the long-term outcomes of regular use of toothpaste containing chlorhexidine and cetylpyridine on the general metabolic control of diabetes.

## Figures and Tables

**Table 1:** The characteristics of examined group *n* = 42.

Variable	Median (IQR)
Age (years)	27 (22–35)
Diabetes duration (years)	12 (9–18)
BMI (kg/m^2^)	23.55 (21.8–26.1)
HbA1c (%)	8.05 (7.1–9.4)
Total cholesterol (mg/dL)	187 (158–202)
LDL-Cholesterol (mg/dL)	85 (70.5–119)
HDL-Cholesterol (mg/dL)	67 (59–79)
Triglycerides (mg/dL)	82 (62–110)
hsCRP (mg/dL)	1.06 (0.39–2.43)

**Table 2:** Comparison of subgroups according to the median value of HbA1c in the study group. The Mann–Whitney U test.

*n* = 42	HbA1c ≤ 8%	HbA1c > 8%	*p*
Age (years)	27 (22–37)	26 (22–35)	0.6
Diabetes duration (years)	13 (6–18)	12 (10–17)	0.9
BMI (kg/m^2^)	23.6 (21.6–27)	23.1 (22–25)	0.9
Total cholesterol (mg/dL)	180 (137–203)	189 (176–195)	0.4
LDL-Cholesterol (mg/dL)	87 (55.5–130.8)	96.4 (86.2–103)	0.4
HDL-Cholesterol (mg/dL)	71 (61–79)	64 (58–74)	0.6
Triglycerides (mg/dL)	74 (48.5–89.5)	99 (71–125)	0.01
hsCRP (mg/dL)	0.83 (0.39–1.5)	2.04 (0.66–3.13)	0.06

**Table 3:** Comparison of clinical indicators of periodontal status before and after the application of the toothpaste. The Wilcoxon signed-rank test.

Before	Median (IQR)	After	Median (IQR)	*p*
API I	0.35 (0.24–0.65)	API II	0.265 (0.18–0.39)	0.03
SBI I	0.07 (0.04–0.15)	SBI II	0.035 (0–0.06)	0.002
GI I	0.88 (0.46–1)	GI II	0.67 (0.25–1)	0.0008

API I: approximal plaque index before the application of the toothpaste, SBI I: sulcus bleeding index before the application of the toothpaste, GI I: gingival index before the application of the toothpaste, API II: approximal plaque index after the application of the toothpaste, SBI II: sulcus bleeding index after the application of the toothpaste, GI II: gingival index after the application of the toothpaste.

**Table 4:** Comparison of clinical indicators of periodontal status before and after the application of the toothpaste in the HbA1c ≤ 8% and HbA1c > 8% subgroups. The Wilcoxon signed-rank test.

HbA1c ≤ 8%				
**Before**	**Median (IQR)**	**After**	**Median (IQR)**	***p***
API I	0.38 (0.23–0.65)	API II	0.25 (0.19–0.4)	0.01
SBI I	0.07 (0.04–1.15)	SBI II	0.04 (0.03–0.07)	0.04
GI I	0.88 (0.48–1)	GI II	0.67 (0.42–1)	0.04
**HbA1c > 8%**				
**Before**	**Median (IQR)**	**After**	**Median (IQR)**	***p***
API I	0.28 (0.24–0.63)	API II	0.29 (0.16–0.39)	0.08
SBI I	0.06 (0.03–0.12)	SBI II	0 (0–0.06)	0.03
GI I	0.83 (0.38–1)	GI II	0.525 (0.25–1)	0.03

**Table 5:** Concentration of selected oxidative stress indicators in saliva before and after the application of the toothpaste. Median (IQR). The Wilcoxon signed-rank test.

	Concentration before	IQR	Concentration after	IQR	*p*
Protein (mg/mL)	2.04	1.6–2.3	1.65	1.5–2.0	0.09
TEAC (nM/mg)	259.9	200.3–306.5	278.1	247.7–334.7	0.009
TBARS (nM/mg)	0.93	0.6–2.6	0.64	0.3–1.1	0.00005
AOPP (nM/mg)	125.8	68.9–195.8	139.0	57.9–310.9	0.052

**Table 6:** Concentration of selected oxidative stress indicators in saliva before and after the application of the toothpaste in the HbA1c ≤ 8% and HbA1c > 8% subgroups. Median (IQR). The Wilcoxon signed-rank test.

	Concentration before	IQR	Concentration after	IQR	*p*
HbA1c ≤ 8%					
Protein (mg/mL)	2.0	1.65–2.15	1.63	1.53–2.04	0.2
TEAC (nM/mg)	266.7	224.2–310.3	283.9	252.5–320.5	0.4
TBARS (nM/mg)	0.95	0.58–2.7	0.42	0.24–0.7	0.002
AOPP (nM/mg)	129.6	112.7–212.5	156.5	59.9–339.6	0.1
	**Concentration before**	**IQR**	**Concentration after**	**IQR**	***p***
HbA1c > 8%					
Protein (mg/mL)	2.07	1.63–2.3	1.73	1.5–1.98	0.4
TEAC (nM/mg)	217.6	191.5–298.0	267.8	245–334.7	0.009
TBARS (nM/mg)	0.9	0.64–2.38	0.89	0.49–1.6	0.02
AOPP (nM/mg)	101.7	36.4–178.0	108.7	49.1–253.2	0.3

## Data Availability

All data is contained within the article.
